# Effects of dietary chlorogenic acid on cecal microbiota and metabolites in broilers during lipopolysaccharide-induced immune stress

**DOI:** 10.3389/fmicb.2024.1347053

**Published:** 2024-03-08

**Authors:** Xiaodi Hu, Wenrui Zhen, Dongying Bai, Jiale Zhong, Ruilin Zhang, Haojie Zhang, Yi Zhang, Koichi Ito, Bingkun Zhang, Yanbo Ma

**Affiliations:** ^1^Department of Animal Physiology, College of Animal Science and Technology, Henan University of Science and Technology, Luoyang, China; ^2^Henan International Joint Laboratory of Animal Welfare and Health Breeding, College of Animal Science and Technology, Henan University of Science and Technology, Luoyang, China; ^3^Department of Food and Physiological Models, Graduate School of Agricultural and Life Sciences, The University of Tokyo, Ibaraki, Japan; ^4^State Key Laboratory of Animal Nutrition, Department of Animal Nutrition and Feed Science, College of Animal Science and Technology, China Agricultural University, Beijing, China; ^5^Longmen Laboratory, Science & Technology Innovation Center for Completed Set Equipment, Luoyang, China

**Keywords:** chlorogenic acid, immune stress, gut microbiota, gut metabolites, broilers

## Abstract

**Aims:**

The aim of this study was to investigate the effects of chlorogenic acid (CGA) on the intestinal microorganisms and metabolites in broilers during lipopolysaccharide (LPS)-induced immune stress.

**Methods:**

A total of 312 one-day-old Arbor Acres (AA) broilers were randomly allocated to four groups with six replicates per group and 13 broilers per replicate: (1) MS group (injected with saline and fed the basal diet); (2) ML group (injected with 0.5 mg LPS/kg and fed the basal diet); (3) MA group (injected with 0.5 mg LPS/kg and fed the basal diet supplemented with 1,000 mg/kg CGA); and (4) MB group (injected with saline and fed the basal diet supplemented with 1,000 mg/kg CGA).

**Results:**

The results showed that the abundance of beneficial bacteria such as Bacteroidetes in the MB group was significantly higher than that in MS group, while the abundance of pathogenic bacteria such as Streptococcaceae was significantly decreased in the MB group. The addition of CGA significantly inhibited the increase of the abundance of harmful bacteria such as Streptococcaceae, Proteobacteria and *Pseudomonas* caused by LPS stress. The population of butyric acid-producing bacteria such as Lachnospiraceae and *Coprococcus* and beneficial bacteria such as Coriobacteriaceae in the MA group increased significantly. Non-targeted metabonomic analysis showed that LPS stress significantly upregulated the 12-keto-tetrahydroleukotriene B4, riboflavin and mannitol. Indole-3-acetate, xanthurenic acid, L-formylkynurenine, pyrrole-2-carboxylic acid and L-glutamic acid were significantly down-regulated, indicating that LPS activated inflammation and oxidation in broilers, resulting in intestinal barrier damage. The addition of CGA to the diet of LPS-stimulated broilers significantly decreased 12-keto-tetrahydro-leukotriene B4 and leukotriene F4 in arachidonic acid metabolism and riboflavin and mannitol in ABC transporters, and significantly increased N-acetyl-L-glutamate 5-semialdehyde in the biosynthesis of amino acids and arginine, The presence of pyrrole-2-carboxylic acid in D-amino acid metabolism and the cecal metabolites, indolelactic acid, xanthurenic acid and L-kynurenine, indicated that CGA could reduce the inflammatory response induced by immune stress, enhance intestinal barrier function, and boost antioxidant capacity.

**Conclusion:**

We conclude that CGA can have a beneficial effect on broilers by positively altering the balance of intestinal microorganisms and their metabolites to inhibit intestinal inflammation and barrier damage caused by immune stress.

## 1 Introduction

In order to meet the growing demand for poultry products, intensive farming methods have been rapidly developed. There are many stress factors in the large-scale breeding factories. Immune stress can be caused by vaccination, improper temperature and humidity, noise, microbial pathogens, etc. (Tiku and Tan, 2021). Immune stress, which results from activation of the immune system by an antigenic stimulus in the environment, directly or indirectly affects animal behavior and metabolism through the neuroendocrine system (Klasing et al., 1987). Immune stress can also negatively alter the composition and balance of the intestinal microbiota, changing the normal physiological and biochemical environment of the intestinal tract, hindering its normal functioning, and leading to a decline in host growth and an increase in the occurrence of disease (Zhang et al., 2021; Ye et al., 2023). Intestinal microbes play key roles in nutritional metabolism, the development of innate immunity, and the prevention of pathogen colonization (Thaiss et al., 2016; Kogut, 2019). Changes in the type and numbers of intestinal bacteria in chickens may adversely affect their feeding efficiency, reduce productivity, lead to intestinal inflammation, and may endanger the digestion, absorption and metabolism of host nutrients (Kohl, 2012). Intestinal bacteria can eliminate pathogens by releasing ligands, such as microbial-related molecular patterns that bind to host cell receptors, to stimulate the immune system (Brisbin et al., 2008), and can also produce antimicrobial peptides to prevent pathogen colonization (Mancabelli et al., 2016). Intestinal microbes produce a variety of bioactive substances and metabolites, such as lipopolysaccharide, peptidoglycan, DNA and extracellular vesicles, short-chain fatty acids (SCFAs), polyamines, secondary bile acids, vitamins, neurotransmitters and phenolic compounds (Zhen et al., 2021; Wang Q. et al., 2023). They can directly or indirectly participate in a variety of physiological processes in health and disease, including host development, metabolism, immune regulation, and intercellular signal communication (Nicholson et al., 2012). Therefore, intestinal microbes and their metabolites are critical to the health of the host.

Antibiotics have long been used to promote the growth of poultry as well as prevent and treat disease (Castanon, 2007). However, antibiotic residues in foods of animal origin can pose health risks to consumers (Suresh et al., 2018) and the overuse of antibiotics can lead to the development of drug-resistant bacteria. Currently, many countries, including China, have banned the use of antibiotics as feed additives, so it is crucial to develop alternatives to antibiotics that can both increase production and keep broilers healthy. Plant extracts with antibacterial properties have been widely developed and tested as safe and effective alternatives to the currently available drugs because their mechanism of action is such that bacterial resistance is unlikely (Chassagne et al., 2020). Recent studies have shown that the effects of some plant extracts depend largely on the regulation of intestinal microbiota (Di Meo et al., 2019; Zhao et al., 2019). Among the active ingredients, chlorogenic acid (CGA), extracted from *Eucommia ulmoides* and *Flos Lonicerae*, has shown promise as an effective substitute for conventional antibiotics in livestock and poultry. CGA has a variety of pharmacological properties, such as antibacterial (Gong et al., 2018), anti-inflammatory, antioxidant (Liang and Kitts, 2015), and the ability to restore homeostasis of the intestinal microbial community (Zhang et al., 2017). Studies have shown that CGA can alleviate intestinal inflammation by inhibiting phosphorylation in the TLR4/NF-κB signaling pathway or the ERK1/2-STAT3 pathway (Chen J. et al., 2018; Vukelić et al., 2018). In addition, CGA can also promote the expression of tight junction proteins by intestinal cells to improve intestinal barrier function (Wu et al., 2018). Our previous studies have also shown that dietary CGA can alleviate the intestinal barrier damage and intestinal inflammation caused by immune stress (Tan et al., 2023). However, the effect of CGA on immune-stressed broilers is still not well understood; therefore, we determined the population of intestinal microorganisms and the microbial metabolite spectrum of immune-stressed broilers fed with chlorogenic acid to investigate whether the effects of CGA on the composition and metabolism of intestinal bacteria could protect against intestinal barrier damage and inflammation.

## 2 Materials and methods

### 2.1 Broilers and experimental treatments

A total of 312 one-day-old healthy male Arbor Acres chickens were obtained from the Henan Quanda Poultry breeding Company. They were randomly divided into four groups with six replicates in each group: (1) MS group, injected with saline and fed the basal diet; (2) ML group, injected with 0.5 mg lipopolysaccharide (LPS)/kg and fed with the basal diet; (3) MA group, injected with 0.5 mg LPS/kg and fed with the basal diet supplemented with 1,000 mg/kg CGA; and (4), the MB group, injected with normal saline and fed the basal diet supplemented with 1,000 mg/kg CGA. The formulation of the basic diet met the requirements of the National Research Council, and the composition of the feed was the same as for the previous experiment (Tan et al., 2023). CGA (98% purity) was purchased from Changsha Staherb Natural ingredients Company (Changsha, China) and thoroughly mixed into the basal diet. Lipopolysaccharide was from Sigma Co. and the serotype of Escherichia coli is O55: B5.

The broilers were housed in three-tier vertical cages equipped with troughs and nipple drinkers to ensure proper feeding and watering, and the cages were cleaned and disinfected regularly. During the first week of the experiment, all broilers were kept at 33°C ± 1°C, and then the temperature was decreased by 1°C per day to 25°C. The relative humidity was 65%−70% during the first week, and then decreased to 50%−65%. Broilers received continuous light in the first 3 days and 23L:1D for the rest of the experiment. All broilers were vaccinated with Newcastle disease-infectious bronchitis vaccine on the 7th day. All the experiments were conducted in accordance with the animal ethics standards and the rules approved by the Animal Protection and Utilization Committee of Henan University of Science and Technology. CGA was added throughout the experiment. Saline or LPS was injected intraperitoneally from the 14th day of age for three consecutive days. At 24 h after the last injection (17 days old), one broiler from each repeat was selected and euthanized. The cecal contents were collected in sterile centrifuge tubes, flash frozen in liquid nitrogen, and stored at −80°C for later analysis.

### 2.2 Gut microbial sequencing

The intestinal contents were sent to Shanghai Personal Biotechnology Co., Ltd. for 16S rRNA gene sequencing. Total microbial DNA was extracted from cecal contents (100 mg) using the QIAamp Rapid Fecal mini-kit (Qiagen, Hilden, Germany). The V3-V4 region of the 16S rRNA gene was amplified by universal primers 338F: ACTCCTACGGGAGGCAGCA and 806R: GGACTACHVGGGTWTCTAAT. PCR products were extracted with a Qiagen gel extraction kit (Qiagen, Germany) and quantified using a Qubit 2.0 fluorometer (Thermo-Fisher, Waltham, USA). High-quality reads were selected from raw sequencing data obtained on an Illumina platform, quality filtered, denoised, spliced and de-chimerized using the DADA2 method. The sequences of each group were compared and annotated using the GreenGenes database. Analysis of α-diversity and β-diversity was performed using Qiime2 (2019.4) software written in Python script. A species diversity matrix was created based on the weighted Bray-Curtis algorithm. Differential abundance of taxa was determined using linear discriminant analysis (LDA) effect size (LEfSe) with *p* < 0.05 and LDA = 2.5 used as thresholds. Functional composition profiles of the gut bacteria were determined from 16S rRNA gene sequences using PICRUSt2 software to count KEGG immediate homologs in Level 2 functional categories. Data were analyzed using the online platform Personalbio GenesCloud (https://www.genescloud.cn). The raw data were uploaded to the National Center for Biotechnology Information's Sequence Read Archive database (SRA accession number: SRP472486:PRJNA1041608).

### 2.3 Non-targeted metabolomics analysis of cecal metabolites

Weighed samples in 2 ml microfuge tubes were suspended in 600 μl of methanol containing 2-chloro-L-phenylalanine (4 ppm), and vortexed for 30 s. Steel beads were added, the tubes were put into a tissue grinder and ground for 120 s at 50 Hz then ultrasound for 10 min at room temperature. The suspensions were centrifuged at 12,000 rpm for 10 min at 4°C, the supernatants were filtered through a 0.22 μm membrane, and the filtrates were transferred into LC-MS vials for analysis. The samples were separated by ultra-high performance liquid chromatography on a ThermoVanquish (Thermo Fisher Scientific, USA) UHPLC system using an Acquity UPLC^®^ HSS T3 column (2.1 × 100 mm, 1.8 μm; Waters, Milford, MA, USA) at a flow rate of 0.3 ml/min and a column temperature of 40°C, with an injection volume of 2 μl. In positive ion mode, the mobile phases were 0.1% formic acid in acetonitrile (B2) and 0.1% formic acid in water (A2). The gradient elution program was as follows: 0–1 min, 8% B2; 1–8 min, 8%−98% B2; 8–10 min, 98% B2; 10.0–10.1 min, 98%~8% B2; 10.1–12.0 min, 8% B2. In negative ion mode, the mobile phases were acetonitrile (B3) and 5 mM ammonium formate in water (A3). The gradient elution program was: 0–1 min, 8% B3; 1–8 min, 8%−98% B3; 8–10 min, 98% B3; 10.0–10.1 min, 98%−8% B3; 10.1–12.0 min, 8% B3. Mass spectrometry was conducted on a Thermo Orbitrap Exploris 120 mass spectrometer (Thermo Fisher Scientific, USA) with electrospray ionization (ESI) source; positive and negative ion modes were used separately to collect data. The positive ion spray voltage was 3.50 kV, the negative ion spray voltage was −2.50 kV, the sheath gas was 40 arb, and the auxiliary gas was 10 arb. The capillary temperature was 325°C, and the primary full scan was performed at a resolution of 60,000, with a primary ion scan range of 100–1,000 in *m*/*z*. The HCD was used for the secondary cleavage with a collision energy of 30% and a secondary resolution of 15,000. The first four ions of the acquired signal were fragmented, while dynamic exclusion was used to remove unnecessary MS/MS information. The original data was converted into mzXML format by ProteoWizard, and peak alignment, retention time correction and peak area extraction were performed by XCMS software. The data extracted by XCMS were identified by metabolite structure identification and data preprocessing, the quality of the experimental data was evaluated, and lastly, the data were analyzed. Data analysis included univariate statistical analysis, multidimensional statistical analysis, differential metabolite screening, and KEGG pathway analysis. All untargeted metabolomic data used in this publication have been deposited to the EMBL-EBI Metabolites database with the identifier MTBLS8986. The complete data set can be accessed at https://www.ebi.ac.uk/metabolights/MTBLS8986.

## 3 Results

### 3.1 Alpha and beta diversity analysis

**Figure 1:**
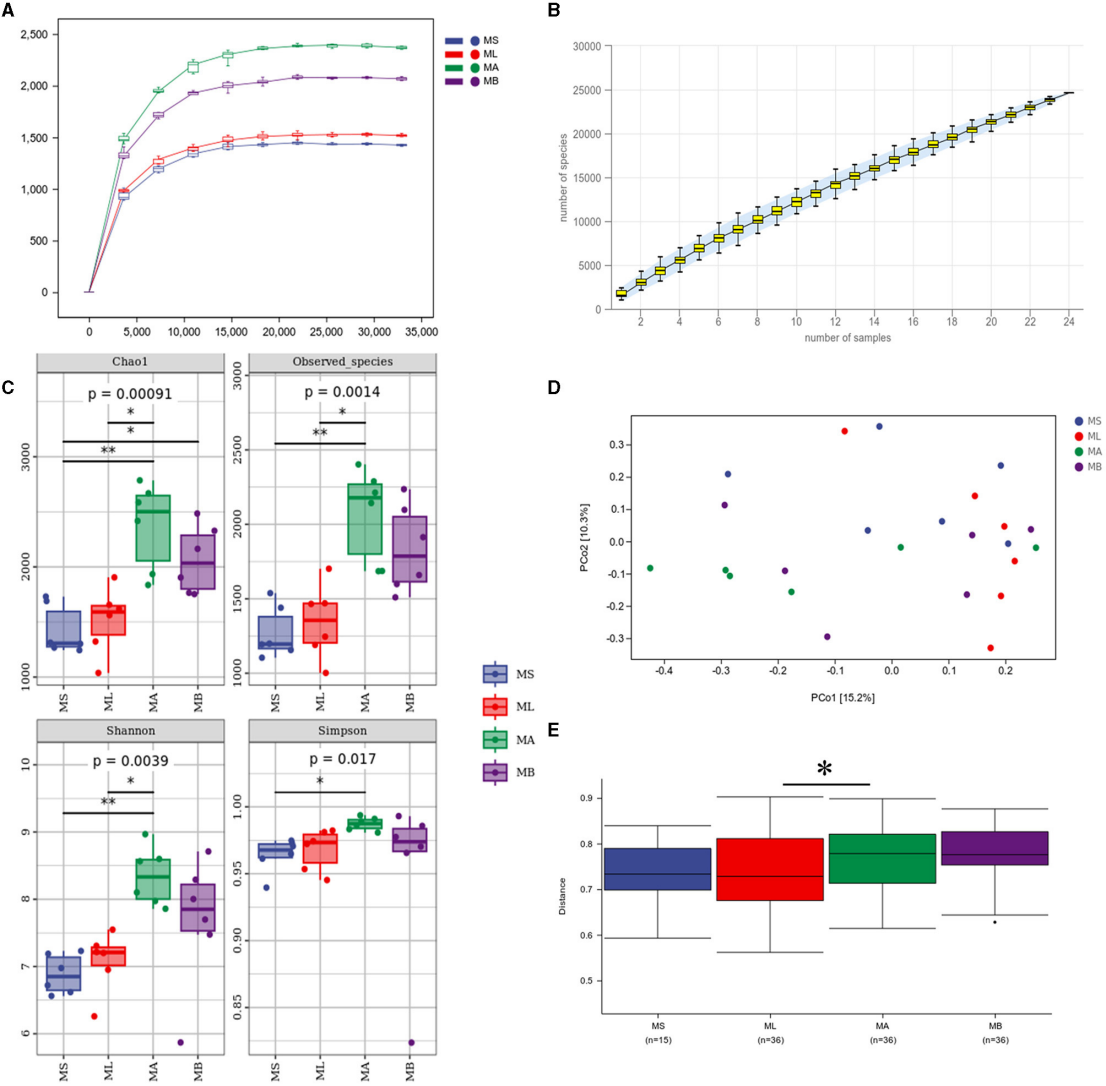
Effect of dietary chlorogenic acid supplementation on cecal microbiota diversity in lipopolysaccharide-challenged broilers. **(A)** Rarefaction curve. **(B)** Species accumulation curves. **(C)** Community diversity and richness. **(D)** Two-dimensional OTU abundance-based principal coordinate analysis (PCoA) of cecal microbiota. **(E)** Analysis of bacterial community structure between groups based on permanova test. *Means significant difference between groups (*p* < 0.05). **Means statistically significant difference between groups (*p* < 0.01).

An average of 70,635 high quality sequences were obtained from each cecal sample. The sparse curve and the species accumulation curve (Figures 1A, B) indicate that the sequencing depth and number of samples were sufficient to adequately reflect the microbial community composition of the cecum. Figure 1C shows the α-diversity and the Chao1 index was significantly higher in the MB group compared with the MS group (*p* < 0.05). The Chao1 index, and the observed species and Shannon indices were significantly higher in the MA group relative to the ML group (*p* < 0.05). Principal coordinate analysis (PCoA) was used to evaluate the similarities and differences between samples and groups. The results showed that the cecal microflora was significantly separated between ML and MA groups (Figure 1D). The weighted bray-curtis distance was used for permanova analysis to quantify the differences in species diversity. The results showed that compared with the ML group, the β diversity index of the MA group changed significantly (Figure 1E).

### 3.2 Analysis of species composition and differences in species between groups

**Figure 2:**
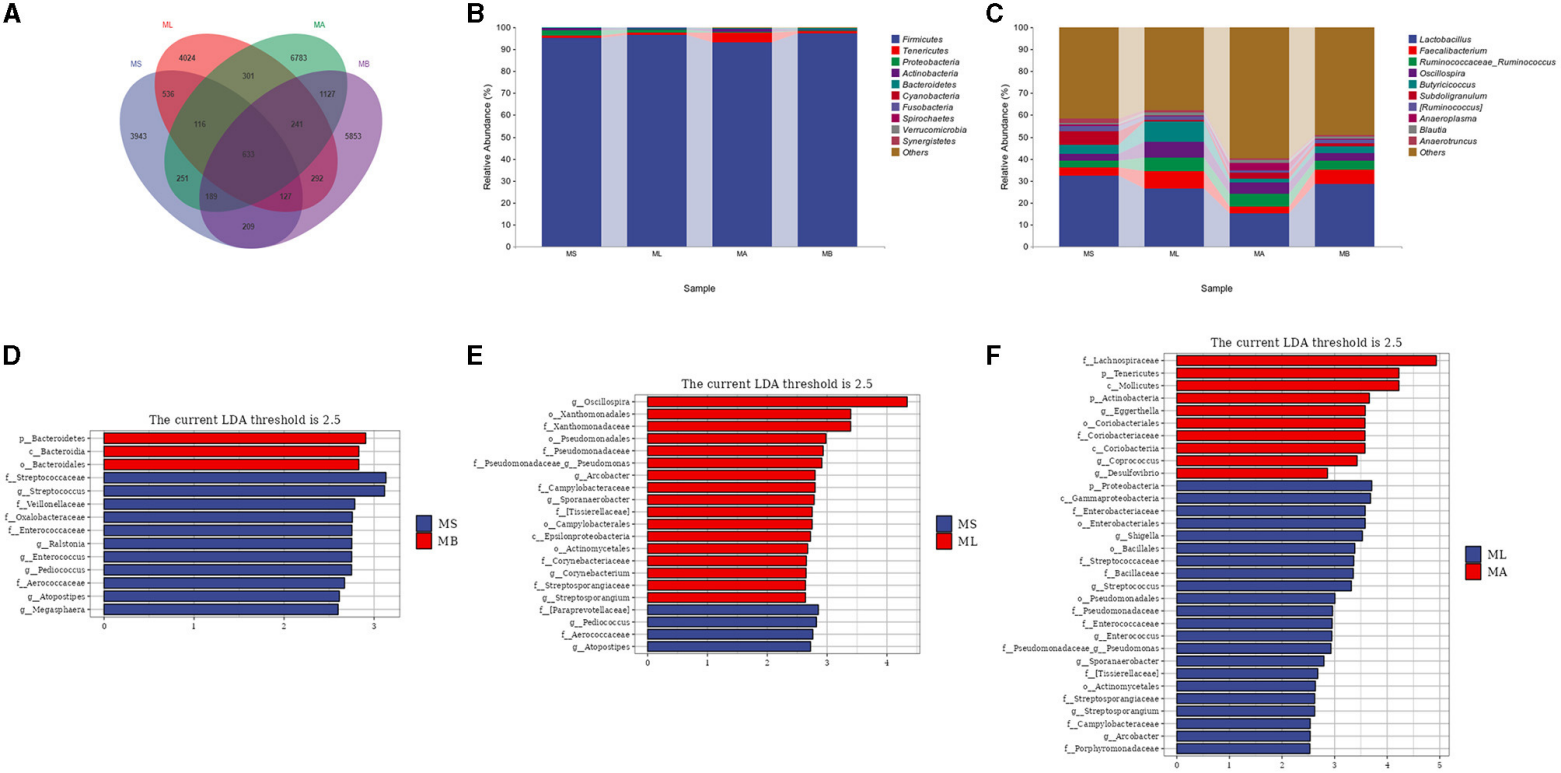
Effect of dietary chlorogenic acid on cecal microbiota composition of lipopolysaccharide-challenged broilers. **(A)** Venn diagram of the composition of bacterial OTUs. **(B)** Microbial composition at the phylum level. **(C)** Microbial composition at the genus level. Differences between the cecal microbiota of MS and MB groups of broilers **(D)**, **(E)** MS and ML groups, and **(F)** ML and MA groups were determined by linear discriminant analysis effect size (LEfSe).

The Venn diagram showed that there were 633 common OTUs in the cecum of each group, while there were 3,943, 4,024, 6,783, and 5,853 unique OTUs in the MS, ML, MA, and MB groups respectively (Figure 2A). Taxonomic composition analysis showed that at the phylum level (Figure 2B), the dominant phyla of the four groups of cecal microorganisms were Firmicutes, Tenericutes, Proteobacteria, Actinobacteria and Bacteroidetes. At the genus level (Figure 2C), the dominant genera of the four groups of cecum microorganisms were *Lactobacillus, Faecalibacterium, Ruminococcaceae_Ruminococcus, Oscillospira, Butyricoccus*, and *Subdoligranulum*. LEfSe analysis showed that the cecal microbiota in the MB group were enriched in Bacteroidetes, Bacteroidia and Bacteroidales, while there were fewer Streptococcaceae, *Streptococcus*, Veillonellaceae, Oxalobacteraceae, Enterococcaceae, *Ralstonia, Enterococcus, Pediococcus*, Aerococcaceae, *Atopostipes*, and *Megasphaera* compared with the MS group (Figure 2D). In contrast to the MS group, the microbiota in the ML group were more abundant in *Oscillospira*, Xanthomonadales, Xanthomonadaceae, Pseudomonadales, Pseudomonadaceae, *Pseudomonas, Arcobacter*, Campylobacteraceae, *Sporanaerobacter*, Tissierellaceae, Campylobacterales, Epsilonproteobacteria, Actinomycetales, Corynebacteriaceae, *Corynebacterium*, Streptosporangiaceae, and *Streptosporangium*, but showed fewer Paraprevotellaceae, *Pediococcus*, Aerococcaceae, *Atopostipes* (Figure 2E). In contrast to the ML group, the cecal microbiota in the MA group were more abundant in Lachnospiraceae, Tenericutes, Mollicutes, Actinobacteria, *Eggerthella*, Coriobacteriales, Coriobacteriaceae, Coriobacteriia, Coprococcus, and Desulfovibrio, but less abundant in Proteobacteria, Gammaproteobacteria, Enterobacteriaceae, Enterobacteriales, *Shigella*, Bacillales, Bacillaceae, Streptococcaceae, *Streptococcus*, Pseudomonadales, Pseudomonadaceae, *Pseudomonas*, Enterococcaceae, *Enterococcus, Sporanaerobacter*, Tissierellaceae, Actinomycetales, Streptosporangiaceae, *Streptosporangium*, Campylobacteraceae, *Arcobacter*, and Porphyromonadaceae (Figure 2F).

### 3.3 Functional prediction of the intestinal microbiota

**Figure 3:**
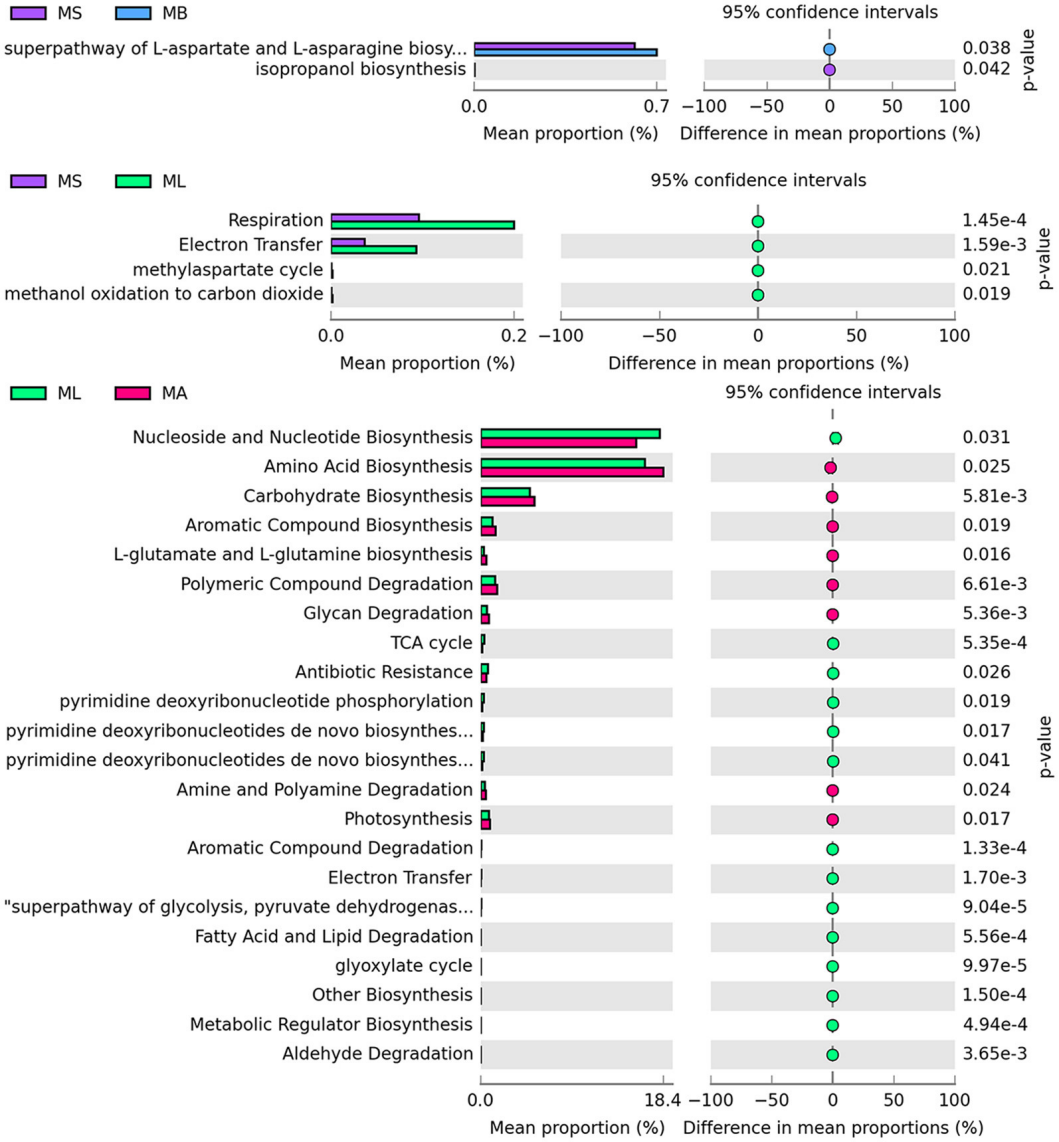
Prediction of microbial function in the broiler cecum. The second level of the KEGG pathway is shown in the extended error bar.

Macrogenomic functions associated with bacterial communities were predicted using PICRUSt2 analysis based on 16S rRNA sequencing data. The differential pathways at the second level of the KEGG analysis are summarized, and the results revealed two differential functional pathways between the MS and MB groups. The MB group was significantly enriched in the superpathway of L-aspartate and L-asparagine biosynthesis. Four differential functional pathways were found between the MS and ML groups. Compared with the MS group, the ML group showed significant enrichment in the functional pathways involved in respiration and electron transfer. Twenty-two differential functional pathways were identified between the ML and MA groups. Fourteen pathways were significantly enriched in the ML group, mainly related to nucleoside and nucleotide biosynthesis, the TCA cycle, electron transfer, and the superpathway of glycolysis. Eight pathways were significantly enriched in the MA group, primarily involved in the biosynthesis of amino acids, carbohydrates, L-glutamate and L-glutamine (Figure 3).

### 3.4 Comparison of differences in intestinal metabolites between groups

**Figure 4:**
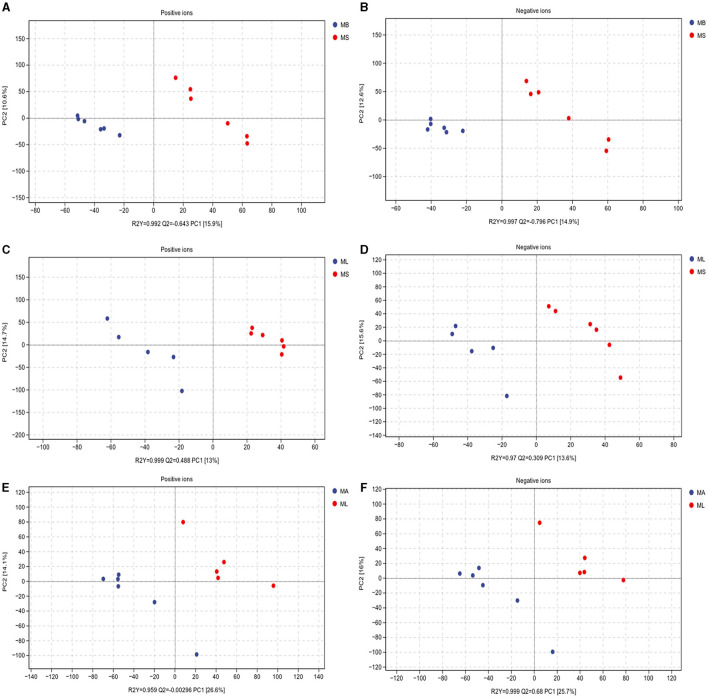
Partial least squares-discriminant analysis (PLS-DA) scores of cecum metabolites. Includes positive and negative ion modes. **(A, B)** Model parameters for MS and MB groups (positive ions, R2Y = 0.992, Q2 = 0.643; negative ions, R2Y = 0.997, Q2 = 0.796). **(C, D)** Model parameters for the MS and ML groups (positive ions, R2Y = 0.999, Q2 = 0.488; negative ions, R2Y = 0.97, Q2 = 0.309). **(E, F)** Model parameters for the ML and MA groups (positive ions, R2Y = 0.959, Q2 = −0.00296; negative ions, R2Y = 0.999, Q2 = 0.68).

To elucidate whether LPS challenge and the addition of CGA affected the intestinal bacteria by altering the intestinal metabolite profile, we evaluated the metabolite composition of the cecal contents using a non-targeted metabolomics approach based on LC-MS/MS. The PLS-DA score plot clearly shows the differences in identified intestinal metabolites between groups, including positive and negative ion patterns. The model parameters of the MS and MB group models are: positive ion model, R2Y = 0.992, Q2 = 0.643; negative ion model, R2Y = 0.997, Q2 = 0.796 (Figures 4A, B). The model parameters of the MS and ML groups are: positive ion model, R2Y = 0.999, Q2 = 0.488; negative ion model, R2Y = 0.97, Q2 = 0.309 (Figures 4C, D). The model parameters of the ML and MA groups are: positive ion model, R2Y = 0.959, Q2 = −0.00296, negative ion model, R2Y = 0.999, Q2 = 0.68 (Figures 4E, F). The results showed that both LPS challenge and CGA could cause significant changes in intestinal metabolites of broilers.

### 3.5 Differential metabolites and metabolic pathways

**Figure 5:**
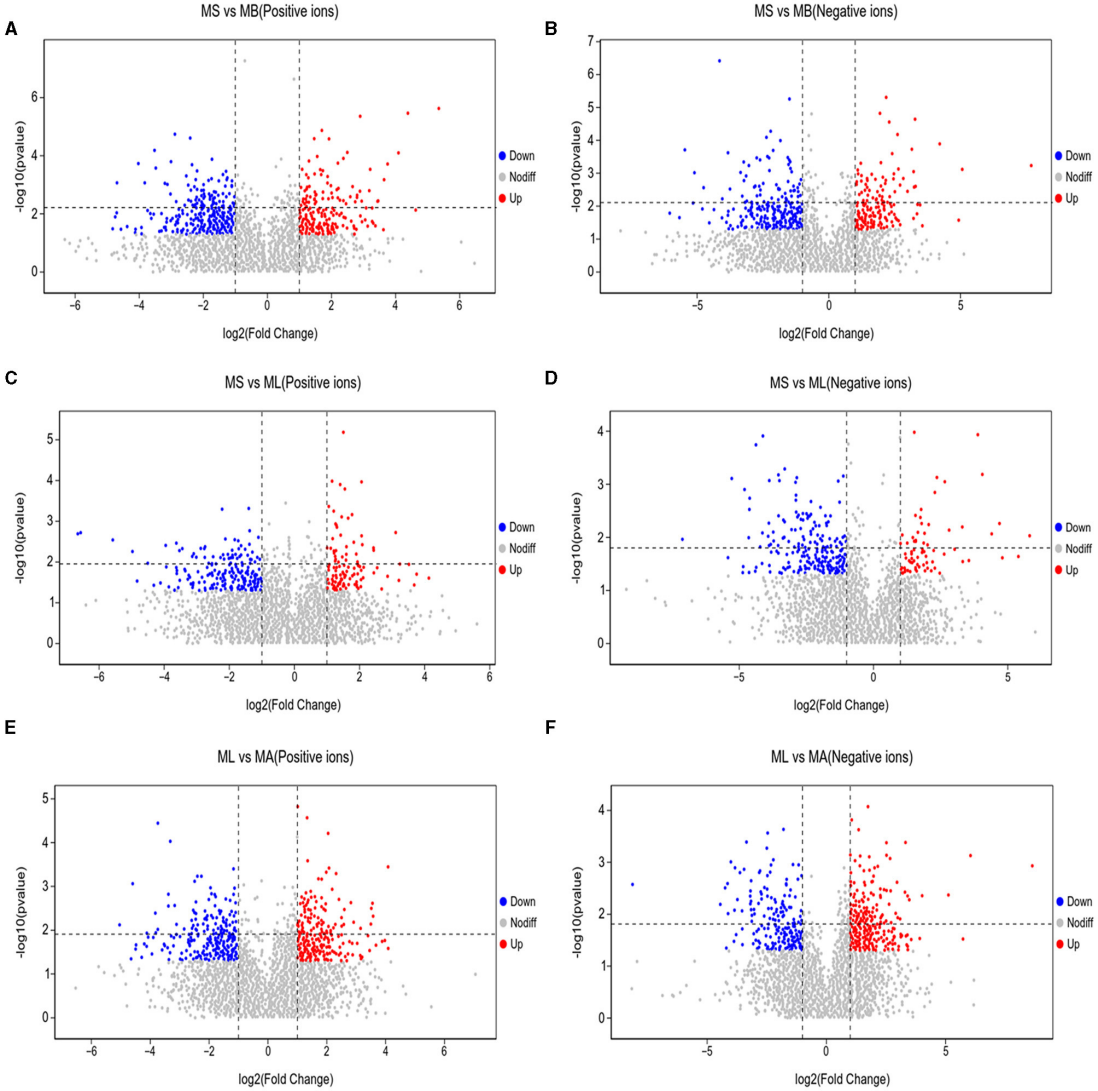
Volcano plots of differential metabolites in the cecum of broilers. Each point represents a kind of metabolite, and the scatter color indicates the final screening result. Significant upregulated metabolites are indicated in red (Up), downregulated metabolites are indicated in green (Down), and nonsignificant differences in metabolites are gray (Nodiff). **(A, B)** Differential metabolite changes in MS and MB groups in positive and negative ion modes. **(C, D)** Differential metabolite changes in MS and ML groups in positive and negative ion modes. **(E, F)** Differential metabolite changes in ML and MA groups in positive and negative ion modes.

**Figure 6:**
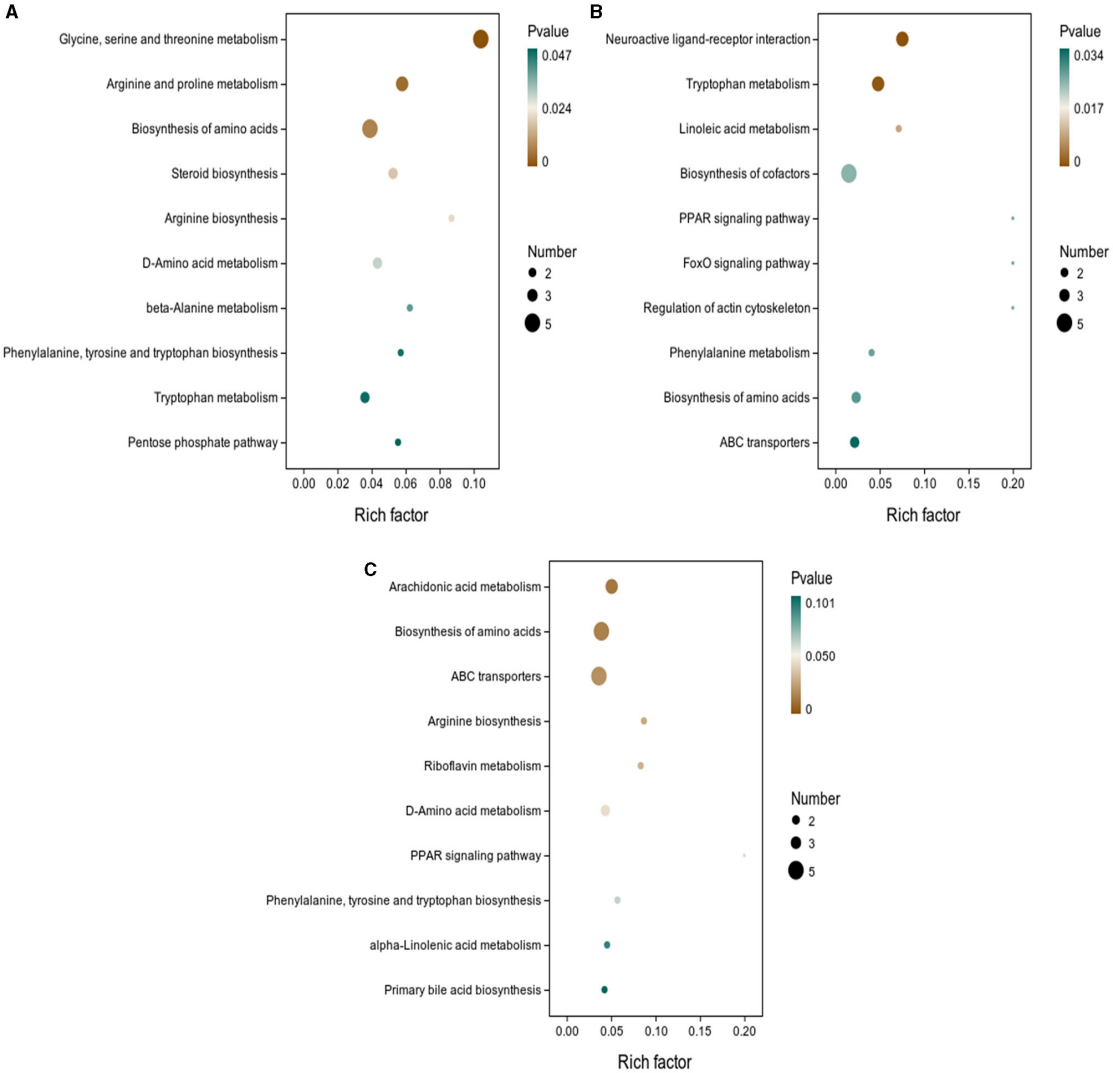
KEGG pathway enrichment analysis of differential metabolites. The enrichment factor is the ratio of the number of differential metabolites annotated to the pathway to all the metabolites annotated to the pathway. The color of the dots represents the *p*-value of the hypergeometric test. KEGG enrichment analysis of differentially expressed metabolites in **(A)** MS and MB groups, **(B)** MS and ML groups, and **(C)** ML and MA groups.

As shown in Figure 5, the results of screening for differential metabolites can be visualized in the form of a “volcano” map. The differences in metabolic pathways are shown in Figure 6 and Tables 1–3. The results showed that dietary CGA significantly altered glycine, serine and threonine metabolism, arginine and proline metabolism, biosynthesis of amino acids, steroid biosynthesis, arginine biosynthesis, D-amino acid metabolism, beta-alanine metabolism, phenylalanine, tyrosine and tryptophan biosynthesis, tryptophan metabolism and the pentose phosphate pathway in normal broilers (Figure 6A; Table 1). Neuroactive ligand-receptor interaction, tryptophan metabolism, linoleic acid metabolism, biosynthesis of cofactors, the PPAR signaling pathway, the FoxO signaling pathway, regulation of the actin cytoskeleton, phenylalanine metabolism, and the biosynthesis of amino acids and ABC transporters were significantly altered in the LPS group compared to the MS group (Figure 6B). Among these, the 12-keto-tetrahydro-leukotriene B4 involved in neuroactive ligand-receptor interactions and the PPAR signaling pathway was significantly up-regulated, while prostaglandin F2 α was significantly down-regulated. Riboflavin and mannitol involved in ABC transporters were significantly up-regulated. L-glutamic acid involved in neuroactive ligand-receptor interaction, biosynthesis of cofactors, the FoxO signaling pathway, biosynthesis of amino acids, ABC transporters, glyoxylate and dicarboxylate metabolism, D-amino acid metabolism and indole-3-acetate, xanthurenic acid, L-formylkynurenine involved in tryptophan metabolism and pyrrole-2-carboxylic acid involved in D-amino acid metabolism were significantly down-regulated (*p* < 0.05; Table 2). Dietary CGA supplementation significantly changed arachidonic acid metabolism, biosynthesis of amino acids, ABC transporters, arginine biosynthesis, riboflavin metabolism, and D-amino acid metabolism in broilers challenged with LPS (Figure 6C). Among them, N-acetyl-L-glutamate 5-semialdehyde enriched in biosynthesis of amino acids and arginine biosynthesis and pyrrole-2-carboxylic acid enriched in D-amino acid metabolism were significantly up-regulated. 12-keto-tetrahydro-leukotriene B4 and leukotriene F4 in arachidonic acid metabolism and riboflavin and mannitol in ABC transporters were significantly down-regulated (*p* < 0.05; Table 3).

**Table 1:** Differential metabolic pathways in the chlorogenic acid supplementation group compared with the control group.

**Pathway ID**	**Pathway name**	***p*-value**	**Up-regulated metabolites**	**Down-regulated metabolites**
gga00260	Glycine, serine and threonine metabolism	0.000	Betaine	2-Ketobutyric acid; glyceric acid; creatine; guanidoacetic acid
gga00330	Arginine and proline metabolism	0.004	N-acetylputrescine; N-succinyl-L-glutamate	Creatine; guanidoacetic acid
gga01230	Biosynthesis of amino acids	0.007	Prephenate; N-a-acetylcitrulline	2-Ketobutyric acid; L-histidine; N-acetylglutamic acid
gga00100	Steroid biosynthesis	0.017	Cholesterol; desmosterol	7-dehydrocholesterol
gga00220	Arginine biosynthesis	0.021	N-a-acetylcitrulline	N-acetylglutamic acid
gga00470	D-Amino acid metabolism	0.029	Linatine	L-histidine; N-acetylglutamic acid
gga00410	beta-alanine metabolism	0.038	Uracil	L-histidine
gga00400	Phenylalanine, tyrosine and tryptophan biosynthesis	0.045	Prephenate; fructose 1,6-bisphosphate	–
gga00380	Tryptophan metabolism	0.046	–	2-aminophenol; L-formylkynurenine; 6-hydroxymelatonin
gga00030	Pentose phosphate pathway	0.047	–	D-ribose; glyceric acid

**Table 2:** Differential metabolic pathways in the ML group compared with the MS group.

**Pathway ID**	**Pathway name**	***P*-value**	**Up-regulated metabolites**	**Down-regulated metabolites**
gga04080	Neuroactive ligand-receptor interaction	0.000	Acetylcholine; 12-keto-tetrahydro-leukotriene B4	L-glutamic acid; prostaglandin F2α
gga00380	Tryptophan metabolism	0.001	3-methoxyanthranilate	Indole-3-acetate; xanthurenic acid; L-formylkynurenine
gga00591	Linoleic acid metabolism	0.009	Linoleic acid; 9,10-DHOME	–
gga01240	Biosynthesis of cofactors	0.024	Riboflavin	L-glutamic acid; pyridoxine; shikimic acid; L-formylkynurenine
gga03320	PPAR signaling pathway	0.026	12-keto-tetrahydro-leukotriene B4	–
gga04068	FoxO signaling pathway	0.026	–	L-glutamic acid
gga04810	Regulation of actin cytoskeleton	0.026	Acetylcholine	–
gga00360	Phenylalanine metabolism	0.027	2-Phenylethanol	N-acetyl-L-phenylalanine
gga01230	Biosynthesis of amino acids	0.028	–	L-glutamic acid; 2-ketobutyric acid; Shikimic acid
gga02010	ABC transporters	0.034	Riboflavin; mannitol	L-glutamic acid
gga00630	Glyoxylate and dicarboxylate metabolism	0.044	–	L-glutamic acid; 4-hydroxy-2-oxoglutaric acid
gga00470	D-amino acid metabolism	0.050	–	L-glutamic acid; pyrrole-2-carboxylic acid

**Table 3:** Differential metabolic pathways in the ML group compared with the MA group.

**Pathway ID**	**Pathway name**	***p*-value**	**Up-regulated metabolites**	**Down-regulated metabolites**
gga00590	Arachidonic acid metabolism	0.012	Prostaglandin F2α; 8,9-DiHETrE	12-keto-tetrahydro-leukotriene B4; leukotriene F4
gga01230	Biosynthesis of amino acids	0.015	Prephenate; N-acetyl-L-glutamate 5-semialdehyde	N-acetylglutamic acid; L-arogenate; N-acetyl-L-2-amino-6-oxopimelate
gga02010	ABC transporters	0.020	Deoxyuridine; phthalic acid	Riboflavin; L-arabinose; mannitol
gga00220	Arginine biosynthesis	0.028	N-acetyl-L-glutamate 5-semialdehyde	N-acetylglutamic acid
gga00740	Riboflavin metabolism	0.030	–	Riboflavin; ribitol
gga00470	D-Amino acid metabolism	0.044	Pyrrole-2-carboxylic acid	N-acetylglutamic acid; 2-oxo-4-methylthiobutanoic acid

## 4 Discussion

Intestinal microbiota plays a key role in host health and the stability of the intestinal environment (Yi et al., 2023). The composition and diversity of the intestinal microbes are essential for maintaining poultry health and production (Kers et al., 2018). A high diversity of intestinal microbiota is beneficial to host health, which is also considered a sign of the maturity of the intestinal population (Turnbaugh et al., 2009). A high level of microbial diversity contributes to the maintenance of a stable intestinal microenvironment against the invasion of pathogenic bacteria when the gut is stressed (Stanley et al., 2014). In this study, alpha diversity analysis showed that dietary CGA significantly increased the cecal microbial species richness (Chao1 index) in healthy broilers and the community diversity (Shannon index) of immune-stressed broilers. The results of PCoA showed that the addition of CGA significantly changed the β diversity index compared with the immune-stressed group. This suggests that dietary CGA could change the cecal microbial community structure of immune-stressed broilers. In agreement with our results, Liu H. et al. (2023) found that CGA treatment significantly increased the Chao1 index and species richness of gut microbes in broilers after dexamethasone-induced immune stress. The community of enriched species enhanced the stability of the intestinal microecology and reduced the susceptibility to bacterial invasion and intestinal inflammation (Ott et al., 2004). Our results demonstrate that dietary CGA can improve the intestinal microbial community of immune-stressed broilers, which appears to be related to intestinal homeostasis.

LEfSe analysis showed that dietary CGA significantly enriched Bacteroidetes, Bacteroidia and Bacteroidales in the ceca of healthy broilers. Bacteroides in the intestinal tract is negatively correlated with intestinal proinflammatory cytokines, which can improve the immune function of the host (Xia et al., 2022). Bacteroides can also maintain the balance of other gut microbes and participate in the synthesis of SCFAs (Chen X. et al., 2018). SCFAs play an important role in maintaining health by preventing disease development and have been shown to increase chemotaxis, phagocytosis, and reactive oxygen species (ROS) production, have anti-inflammatory, anti-tumor and antibacterial effects, improve intestinal integrity, and maintain intestinal homeostasis and optimal functioning of the immune system (Metzler-Zebeli et al., 2021). Other studies have shown that CGA promoted an increase in the relative abundance of Bacteroidaceae (Wang Z. et al., 2019), which is similar to our results. Yan et al. (2022) showed that CGA not only improved the α- and β-diversity of bacteria in the cecum, but also specifically increased the abundance of Bacteroidetes. Dietary supplementation with CGA significantly decreased the relative abundance of Streptococcaceae, *Streptococcus*, Veillonellaceae and Enterococcaceae in the ceca of healthy broilers. Some *Streptococci* are pathogens that can cause respiratory and gastrointestinal diseases in broilers (Png et al., 2010). Tsay et al. (2018) revealed that exposure of airway epithelial cells to *Streptococcus* and *Veillonella* up-regulated ERK and PI3K signaling pathways and led to disease. Enterococcaceae are known as conditional pathogens, because they can cause enteritis in Toll-like receptor 5 (TLR5)-deficient mice (Horne et al., 2019). Enterococcaceae are often associated with simple fever and diarrhea, but in some cases the infection is more serious (Hakim et al., 2018). The above results showed that the addition of CGA to the diet favored the growth of beneficial bacteria such as Bacteroidetes, Bacteroidia and Bacteroidales, and inhibited the growth of pathogenic or conditionally pathogenic bacteria such as Streptococcaceae, *Streptococcus*, Veillonellaceae, and Enterococcaceae, which indicated that the addition of CGA could improve the balance of gut microbes and the health of broilers.

LPS challenge significantly enriched the microbial population of Pseudomonadales, Pseudomonadaceae, *Pseudomonas, Arcobacter*, Campylobacteraceae, Campylobacterales, and Epsilonproteobacteria in the ceca of broilers, which belong to the pathogenic bacterial group of Proteobacteria. The massive abundance of Proteobacteria in the gut reflects dysbiosis or instability in the structure of the gut microbial community (Shin et al., 2015). *Pseudomonas* is a conditionally pathogenic bacterium that is more abundant in the gut of patients with inflammatory bowel disease (Wagner et al., 2008). *Pseudomonas aeruginosa* can induce an inflammatory response by activating inflammatory cells (Wang Q. et al., 2019). *Arcobacter* is a new genus of zoonotic foodborne and waterborne pathogens that can cause diarrhea, bacteremia and other diseases in humans and animals (Hänel et al., 2016). It has been shown that some species of *Arcobacter* can adhere to and invade eukaryotic cells, induce immune responses and produce toxins that damage host cells (Ferreira et al., 2016). *Campylobacter* is considered to be a zoonotic genus and is the main pathogenic factor of bacterial gastroenteritis (Rao et al., 2021). *Campylobacter* infection can cause various complications, such as bacteremia, peritonitis, arthritis and hepatitis (Peterson, 1994; Pope et al., 2007). Corynebacteriaceae, *Corynebacterium* and *Oscillospira* were also significantly enriched in the immune-stressed group, and some *Corynebacterium* species are dangerous pathogens for humans and animals (Tauch et al., 2016). *Oscillospira* is an understudied genus of anaerobic bacteria in the Clostridiaceae family that has been reported to be positively associated with gallstones, diabetes and Parkinson's disease (Goodrich et al., 2014; Vascellari et al., 2020). In summary, the above results indicate that LPS stimulation can cause an imbalance in the intestinal microbiota and bacterial translocation in broilers. This can promote the proliferation of harmful bacteria, alter the normal physiological and biochemical environment of the intestinal tract, and negatively affect its functioning. An imbalance in the proportion of beneficial bacteria relative to pathogens results in inflammatory responses and diseases in the intestinal tract, which can have severe impacts on the health and production of poultry.

However, the supplementation of CGA in broiler diets under LPS challenge significantly reduces the abundance of Streptococcaceae, *Streptococcus*, Enterococcaceae, *Enterococcus*, Proteobacteria, Pseudomonadales, Pseudomonadaceae, *Pseudomonas*, Campylobacteraceae, *Arcobacter* and other pathogenic bacteria, supporting the hypothesis that CGA can significantly reverse the proliferation of multiple types of pathogenic bacteria in the intestinal tract of broilers under immune stress. The addition of CGA to the diet of LPS-challenged broilers, significantly enriched cecal populations of Lachnospiraceae, *Eggerthella*, Coriobacteriales, Coriobacteriaceae, Coriobacteriia, *Coprococcus* and *Desulfovibrio*. *Eggerthella* can promote gastrointestinal inflammation and is related to the consumption of butyric acid (Simpson et al., 2021), while Lachnospiraceae and *Coprococcus* are involved in the production of butyric acid (Miquel et al., 2014; Manrique Vergara and González Sánchez, 2017). It has been shown that Coriobacteriales and Coriobacteriia are positively correlated with acetic acid levels (Xu et al., 2021). Acetic acid can provide energy for the production of butyric acid from acetic acid through the CoA transferase pathway (Duncan et al., 2002). Some studies have shown that butyric acid plays a beneficial role in maintaining the integrity of the intestinal barrier and preventing inflammation, and it has been suggested that the addition of CGA may promote the dynamic balance of intestinal butyric acid-producing bacteria (Medvecky et al., 2018; Zou et al., 2019; Xie et al., 2021). Moreover, butyric acid treatment reversed the LPS-induced cellular inflammatory response, which was positively associated with feed conversion and growth in broilers (Stanley et al., 2016; Wang X. et al., 2023). Some studies have shown that the combination of CGA and epigallocatechin gallate produced an increase in the proportion of Lachnospiraceae, improving the integrity of the intestinal barrier and maintaining intestinal permeability in aged mice (Wei et al., 2023). Butyric acid is also an important chemical in the gut-brain axis, studies have shown that *Clostridium butyricum* treatment could reduce microglia-mediated neuroinflammation through the gut-brain axis via the mediation of butyric acid. Butyric acid treatment of BV2 cells reduced the level of cyclooxygenase-2 (COX-2) and inhibited NF-κB p65 phosphorylation (Sun et al., 2020). NF-κB is essential for the induction of the COX-2 gene, and inhibiting NF-κB can reduce the activity of COX-2 and the synthesis of PGE2. COX-2 is a key messenger molecule in the process of inflammation, which mediates the production and release of a series of inflammatory factors such as PGE2, TNF-α and IL-6 (Bellik et al., 2012; Wan et al., 2018). Furthermore, in a previous study (Liu K. et al., 2023), we found that immune stress mediates growth inhibition in broilers by regulating MAPK-NF-κB signal pathway and activating COX-2-PGE2-EP4 signal axis, and this growth inhibition can be reversed by blocking COX-2 activity. There is some evidence that the increased abundance of Coriobacteriaceae may be related to the inhibition of COX-2 activity (Montrose et al., 2016). Thus, CGA may alleviate the LPS-induced inflammatory response, intestinal barrier damage, and growth inhibition by inhibiting COX-2 activity and altering signaling through the gut-brain axis.

Dietary CGA could also increase the relative abundance of cecal *Desulfovibrio* in immune-stressed broilers. *Desulfovibrio* has been associated with inflammatory bowel disease (Rowan et al., 2010) and metabolic diseases (Petersen et al., 2019), but it is not always related to adverse health effects. Some researchers have shown that the relative abundance of *Desulfovibrio* is positively correlated with microbial community diversity, which is beneficial to microbiome stability and host health (Le Chatelier et al., 2013). *Desulfovibrio* has a positive correlation with *Coprococcus* and a negative correlation with *Streptococcus* (Chen et al., 2021), which is consistent with our results. Thus, CGA may ameliorate LPS-mediated gut inflammation and barrier damage by decreasing the abundance of a number of harmful species of bacteria and increasing the numbers of butyric acid-producing bacteria such as Lachnospiraceae and *Coprococcus*, as well as beneficial bacteria in the class Coriobacteria, such as Coriobacteriales and Coriobacteriaceae that can influence host physiology through the microbial gut-brain axis.

Predictive analysis by PICRUSt2 showed that addition of CGA to healthy broilers significantly enriched the superpathway of L-aspartic acid and L-asparagine biosynthesis. Aspartic acid (Asp) is an important component of protein synthesis, and more specifically the precursor of key neurotransmitters and physiological regulators (Errico et al., 2009). Asp can also regulate immune function as the precursor for asparagine (Asn) synthesis (Wu and Meininger, 2002). Asn not only stimulates the proliferation of intestinal mucosal cells, but also enhances the metabolism of the body under aerobic conditions and increases energy production (Marquezi et al., 2003). Therefore, the dietary addition of CGA could have a protective effect on the intestinal environment of healthy broilers. The immune-stressed group showed significant enrichment in functional pathways such as respiration, the glycolysis superpathway, the TCA cycle, electron transfer, nucleoside and nucleotide biosynthesis. Glycolysis, the tricarboxylic acid cycle, and the electron transport chain are the three stages of respiration. The increase in mitochondrial respiration rate is often accompanied by the generation of large amounts of reactive oxygen species (ROS) (Oshima et al., 2004). The mitochondrial biofilm system is rich in unsaturated fatty acids, which can be peroxidized by ROS to generate lipid peroxides, resulting in disruption of the biofilm structure. It has been shown that *Salmonella* infection induced up-regulation of glycolysis and amino acid catabolism, leading to energy and amino acid depletion in chickens (Kogut and Arsenault, 2017). Nucleotides have also been used as warning or risk-related molecular patterns in stressful environments (Coutinho-Silva and Savio, 2021). In the case of homeostasis disorder, extracellular nucleotides can trigger the production and release of ROS and reactive nitrogen species (RNS) (Alves et al., 2020). Our results suggest that immune stress may lead to intestinal homeostasis disorder and loss of redox balance in broilers exposed to LPS. CGA addition significantly enriched the biosynthetic pathways of amino acids, carbohydrates, L-glutamate, and L-glutamine. Carbohydrates can be fermented by intestinal microbes into short-chain fatty acids, which are the main source of energy for intestinal epithelial cells; they also protect the host against inflammation, fight intestinal diseases, and improve the intestinal barrier function (Fukuda et al., 2011). L-glutamic acid has a variety of biological functions such as participating in protein synthesis, nitrogen, glucose and energy metabolism, and acting as a neurotransmitter (Galland et al., 2017). L-glutamic acid is an important precursor of several conditionally essential amino acids, such as glutamine, proline and arginine, and the glutathione produced by L-glutamic acid plays an important role in the protection of intestinal mucosa from oxidation (Reeds et al., 2000). Glutamine is a source of carbon and reduced nitrogen in many biosynthetic reactions and controls redox homeostasis (Son et al., 2013). Prior research has shown that N-acetyl-L-glutamic acid accelerates early intestinal development in chickens by enhancing intestinal immunity and absorption, thereby increasing the efficiency of feed utilization (Wang et al., 2020). The above results emphasize how effective the addition of CGA to the diet of broiler chickens is in alleviating intestinal inflammation caused by immune stress and protecting the intestinal barrier from oxidative damage.

Many microbial metabolites are biologically active and affect the differentiation, migration, proliferation and apoptosis of host cells, resulting in a variety of physiological or pathological effects to the host. In our experiments, the addition of CGA to the diet of broilers significantly changed the pathways for the metabolism of glycine, serine, threonine, arginine, proline, D-amino acids, beta-alanine, and tryptophan and the biosynthesis of amino acids, arginine, phenylalanine, tyrosine and tryptophan, and the pentose phosphate pathway. Amino acids are essential for the growth and development of animals, biological regulation, coordination and defense (Watford and Wu, 2018). Glycine, serine and threonine play key roles in regulating immune function, inflammatory reactions, microecological balance and antioxidant defense (Chen et al., 2017; Newman and Maddocks, 2017; Rom et al., 2020). Arginine and its metabolites are essential for tissue remodeling, growth and development and immune regulation in mammals (Flynn et al., 2002). Proline and its metabolites are needed as scavengers of oxygen free radicals and regulators of the redox balance (Phang et al., 2010). D-amino acids help to maintain an ecological balance of beneficial bacteria and resist external environmental pressure (Alvarez et al., 2018). Beta-alanine can increase carnosine content, improve growth, reduce tissue oxidation, and increase meat quality in poultry production (Łukasiewicz et al., 2015). Phenylalanine (Phe) is mainly involved in the metabolism of fat and carbohydrates in animals and is converted to tyrosine under the action of Phe hydroxylase, providing a precursor for the synthesis of hormones and neurotransmitters (Oliphant and Allen-Vercoe, 2019). Tryptophan and its metabolites reduce oxidative stress, and modulate immune function and systemic homeostasis (Liu et al., 2015). Given that amino acids and their metabolites play an important role in the physiological function of the body, we speculate that CGA may affect the health of broilers by changing the synthesis and metabolism of intestinal amino acids.

In our experiment, LPS treatment up-regulated 12-keto-tetrahydro-leukotriene B4 and down-regulated prostaglandin F2α in the pathway for the interaction between neuroactive ligands and receptors. The signaling molecule, 12-keto-tetrahydro-leukotriene B4 belongs to the leukotrienes (LTs) family, which is a group of inflammatory mediators produced by the metabolism of arachidonic acid (AA) through the 5-lipoxygenase (5-LOX) pathway (Bernström and Hammarström, 1982). LTs are a powerful class of chemotactic molecules that regulate the migration and activation of leukocytes (Moore et al., 1991). The main function of LTB4 is to activate and recruit neutrophils and macrophages, which have potent biological activities (Lee et al., 2018). Numerous studies have shown that LTB4 is mainly involved in the immune response through inflammation, which plays a significant role in a variety of diseases (Miyabe et al., 2017). LOX secreted by pathogens can initiate the conversion of AA to leukotriene B4, which leads to inflammation (Boutet et al., 2003). PGF2α is obtained from AA via the COX-2 pathway to PGH2, and is produced by PGH2 metabolism. When the LOX pathway is activated, AA may be converted to leukotrienes, which reduce the synthesis of prostaglandins (Lin and O'Connor, 2014). In this study, we found that LPS altered the dynamic balance of inflammatory mediators and caused the occurrence and development of physiological and pathological processes. In addition, LPS-induced immune stress also led to a significant up-regulation of riboflavin and mannitol in ABC transporters. Riboflavin plays an important role in lipid metabolism, energy metabolism, anti-inflammation and antioxidation, which are essential for maintaining health and preventing diseases (Suwannasom et al., 2020). Interference with intestinal transport, including disorders of digestion and absorption, can hinder the absorption and utilization of riboflavin (Pinto and Zempleni, 2016). In some pathological or stress states, the intestinal mucosa is damaged and atrophied, and the intestinal absorption area is reduced, which leads to decreases in the mannitol flux, destruction of the tight junctions between intestinal epithelial cells, and an increase in intestinal permeability (van der Hulst et al., 1998). In our experiments, riboflavin and mannitol were significantly up-regulated, indicating that LPS caused intestinal damage, problems with absorption dysfunction, and intestinal barrier deterioration in broilers. However, the feeding of dietary CGA to LPS-stimulated broilers significantly down-regulated 12-keto-tetrahydro-leukotriene B4, leukotriene F4 involved in arachidonic acid metabolism and riboflavin and mannitol involved in ABC transporters. The results showed that dietary CGA could reduce the inflammatory response induced by LPS, alleviate the stress-induced damage to the intestinal barrier, improve digestion, absorption, and intestinal health.

LPS stimulation significantly down-regulated indole-3-acetate, xanthurenic acid, and L-formylkynurenine in the tryptophan metabolism of broiler cecum contents in our study. Tryptophan catabolism is considered to be an important factor in inflammation and immune response (Platten et al., 2019), and tryptophan plays a positive role in reducing intestinal permeability and pro-inflammatory cytokine expression in irritable bowel syndrome (Liu Y. et al., 2017). Tryptophan is involved in three metabolic pathways, kynurenine, 5-hydroxytryptamine and indoleacetic acid (Cheng et al., 2018). Xanthurenic acid and L-formylkynurenine are kynurenine metabolites in animals. The products of kynurenine metabolism improve the body's immune system mainly by regulating the production of immunoglobulins and lymphocytes (Zhu, 2010). Gao et al. (2018) found that L-tryptophan and its endogenous metabolites, such as indole and its derivatives, are important nutrients in mammals, have been implicated in intestinal immune homeostasis and a variety of immune disorders, and are able to increase the production of anti-inflammatory cytokines, thereby reducing inflammation. It has been suggested that decreasing levels of indole and its derivatives may activate the inflammatory response and increase intestinal permeability (Xue et al., 2023). The results suggest that LPS-induced immune stress may activate intestinal inflammation and increase intestinal permeability by reducing the content of indole-3-acetate, xanthurenic acid, L-formylkynurenine and other metabolites in the tryptophan metabolic pathway. In contrast, the addition of CGA to the diet of broilers under LPS-induced immune stress could significantly up-regulate indolelactic acid, xanthurenic acid and L-kynurenine in the cecum, showing that CGA can improve the inflammatory response and maintain intestinal barrier function by altering the levels of metabolites in the tryptophan metabolic pathway.

Broilers in the immune stress group showed significant down-regulation of L-glutamic acid involved in neuroactive ligand-receptor interactions, biosynthesis of cofactors, the FoxO signaling pathway, biosynthesis of amino acids, ABC transporters, glyoxylate and dicarboxylate metabolism and D-amino acid metabolism and pyrrole-2-carboxylic acid involved in D-amino acid metabolism. L-glutamic acid is an important precursor of several conditionally essential amino acids, such as glutamine, proline, and arginine, which provide energy to the intestine, reduce mucosal damage caused by bacterial endotoxin, delay apoptosis of lymphocytes, and promote cell growth (Wang et al., 2015), and produce glutathione which is important in intestinal redox homeostasis (Reeds et al., 2000). Pyrrole-2-carboxylic acid is a common metabolite in many organisms, which can be derived from the oxidation of hydroxyproline by D-amino acid oxidase (Bouthillier and Letellier, 1956). Pyrrole-2-carboxylic acid has antibacterial, anti-inflammatory, anti-tumor and other biological activities (Komiyama et al., 1986; Banwell et al., 2006). Some studies have shown that an increase in oxidative stress caused the significant decrease in pyrrole-2-carboxylic acid (Jin et al., 2014). Researchers also found that pyrrole-2-carboxylic acid is a selective inhibitor of 4-hydroxyproline-2-epimerase (Hy PRE), and Hy PRE is an important enzyme expressed in various pathogenic bacteria, including *Pseudomonas* (Gryder and Adams, 1969; Adams, 1973). Therefore, the significant down-regulation of L-glutamic acid and pyrrole-2-carboxylic acid indicated that immune stress decreased intestinal antioxidant capacity and increased levels of pathogenic bacteria in broilers. However, the addition of CGA to the diet of immune-stressed broilers significantly increased the levels of N-acetyl-L-glutamate 5-semialdehyde involved in biosynthesis of amino acids and arginine biosynthesis and pyrrole-2-carboxylic acid involved in D-amino acid metabolism. N-acetyl-L-glutamate 5-semialdehyde is an intermediate in glutamate metabolism, which is consistent with the predicted results of PICRUSt2 in this study. As a precursor of arginine synthesis, N-acetyl-L-glutamate 5-semialdehyde participates in the arginine biosynthetic pathway. Arginine has been proved to play an important role in regulating and protecting intestinal mucosal barrier function and relieving oxidation and inflammation (Liu G. et al., 2017). Therefore, the significant increase in N-acetyl-L-glutamate 5-semialdehyde and pyrrole-2-carboxylic acid in broilers on the diet supplemented with CGA may reduce the oxidative damage caused by immune stress and retard the increase in pathogenic bacteria such as *Pseudomonas*.

## 5 Conclusions

In conclusion, the addition of CGA to the diet can increase the abundance of beneficial bacteria such as Bacteroidetes in the ceca of normal broilers, reduce the abundance of pathogenic bacteria such as Streptococcaceae, affect the synthesis and metabolism of intestinal amino acids, and exert a probiotic function. The addition of CGA could inhibit the increase in harmful bacteria such as Streptococcaceae and Proteobacteria caused by LPS stress, and significantly increase the abundance of butyric acid-producing bacteria such as Lachnospiraceae and *Coprococcus*, and beneficial bacteria such as Coriobacteriia. Altering the levels of 12-keto-tetrahydro-leukotriene B4, leukotriene F4, xanthurenic acid and other metabolites in the ceca of broilers increase the anti-inflammatory, antioxidant and probiotic effects, which may be one of the mechanisms by which CGA reduces intestinal inflammation and barrier damage in immune-stressed broilers.

## Data availability statement

The data presented in the study are deposited in the https://www.ncbi.nlm.nih.gov/, accession number is PRJNA1041608 and https://www.ebi.ac.uk/metabolights/, accession number is MTBLS8986.

## Ethics statement

The animal study was approved by Animal Care and Use Committee of Henan University of Science and Technology. The study was conducted in accordance with the local legislation and institutional requirements.

## Author contributions

XH: Data curation, Formal analysis, Investigation, Methodology, Software, Writing—original draft, Writing—review & editing. WZ: Formal analysis, Software, Writing—original draft, Writing—review & editing. DB: Formal analysis, Writing—review & editing. JZ: Data curation, Investigation, Methodology, Software, Writing—review & editing. RZ: Data curation, Investigation, Writing—review & editing. HZ: Data curation, Methodology, Writing—review & editing. YZ: Formal analysis, Writing—review & editing. KI: Writing—review & editing. BZ: Writing—review & editing. YM: Project administration, Supervision, Writing—review & editing.
